# The effect of the online flipped classroom on self-directed learning readiness and metacognitive awareness in nursing students during the COVID-19 pandemic

**DOI:** 10.1186/s12912-022-00804-6

**Published:** 2022-01-19

**Authors:** Safoura Khodaei, Shirin Hasanvand, Mohammad Gholami, Yaser Mokhayeri, Mitra Amini

**Affiliations:** 1 Student Research Center, Lorestan University of Medical Sciences, Khorramabad, Iran; 2 School of Nursing and Midwifery, Lorestan University of Medical Sciences, Khorramabad, Iran; 3 Cardiovascular Research Center, Shahid Rahimi Hospital, Lorestan University of Medical Sciences, Khorramabad, Iran; 4 Clinical Education Research Center, Shiraz University of Medical Sciences, Shiraz, Iran

**Keywords:** Flipped classroom, Online learning, Self-directed learning, Metacognitive awareness, Nursing students, COVID-19, Learning Management System

## Abstract

**Background:**

The COVID-19 pandemic has initiated digital developments in higher education while closing in-person university classes. As this crisis continues, the need to revive virtual learning opportunities was seriously felt. The present study was conducted to determine the online flipped classroom’s effect on nursing students’ self-directed learning readiness and metacognitive awareness.

**Methods:**

This quasi-experimental single-group study with pretest-posttest design recruited 34 sophomore students of a nursing school in Lorestan province, Western Iran selected by census according to the inclusion criteria. Online asynchronous learning and online flipped classrooms were used during the semester’s first and second eight weeks, respectively. Students filled out self-directed learning readiness scale and metacognitive awareness inventory online before, in the middle of, and at the end of the semester. Data were analyzed using paired t-test in Stata-14 software.

**Results:**

There was no significant difference between the mean score of metacognitive awareness before and after Online asynchronous learning (P=0.15), but the mean score of self-directed learning readiness increased significantly after OA (P=0.0004). After applying online flipped classrooms, students’ mean (SD) scores of metacognitive awareness and self-directed learning readiness were 272.03 (53.03) and 162.03 (21.77), respectively, which confirmed their significant improvement compared to before the intervention. A comparison of the mean score changes of both methods indicated that their implementation did not lead to significant differences between the mean total score of metacognitive awareness (P=0.15) and the mean total score of self-directed learning readiness (P=0.07).

**Discussion:**

Online flipped classroom approach can be used as an effective method in nursing education by improving self-directed learning and metacognitive awareness, which are essential in online education for nursing students.

## Background

The COVID-19 pandemic has initiated digital developments in higher education and closed in-person classes in universities and schools [1–3]. The continued crisis raised the need to revitalize virtual learning opportunities. UNESCO recommended using distance learning and training programs and open access platforms to reduce learning process disruption [4]. Hence, online learning methods were introduced as appropriate educational strategies during the COVID-19 pandemic [1]. Such a sudden shift in educational approach from traditional to online learning without sufficient opportunity to prepare and design virtual classes created some challenges [1, 5]. Many teachers and students were accustomed to physical classes, while all theoretical classes became online [6]. Most teachers have to make rapid advances in information technology literacy as online learning managers and use new teaching methods to increase learners’ willingness [7]. Distance education also requires independent students, i.e., self-motivated learners [8]. Attitude and motivation are considered the main predictors of the desire to continue education for any type of technology-based education [9]. According to a study, online learning requires sufficient mental, physical, and economic readiness to support students’ learning defects [10] especially, when some students’ access to the Internet is poor, they need to be empowered to engage in active self-disciplined, and self-directed learning [11].

Hence, self-directed learning is an essential dimension [12]. Self-directed learners are empowered to decide the information they want to master [13]. Furthermore, metacognitive skills are the first critical components of the skills needed for self-directed learning [10]. In other words, there is a connection between self-directed learning and metacognition [14]. A study has pointed out a positive and significant relationship between metacognitive skills and self-directed learning. Metacognition is an essential part of the learning process defined as awareness and control of learning with two components of cognition, i.e. awareness and regulation of thinking and metacognition regulation or the management of the thinking process [15, 16].

It appears that computer-based educational environments and problem-based learning increase metacognition and self-direction, and the blended approach of flipped classroom model motivates students for self-directed learning and promotes their metacognitive awareness [13, 17]. The effect of flipped classrooms as an active blended educational method on promoting self-directed learning and metacognition has been approved in several studies [18–20].

The online flipped classrooms(OFC) model, a pedagogical approach [21], is among the most popular educational methods during the COVID-19 epidemic and a promising alternative for teaching theoretical courses [22]. In traditional flipped classrooms, learners are persuaded to view video lectures at home and prepare for joint meetings. However, unlike the original flipped classroom model, learners and instructors do not meet physically but online [21]. Using social media platforms for counseling in parallel with the flipped online classroom in this unexpected crisis is critical [23]. As a widely recognized, effective, innovative, and significant strategy in the higher education of different countries, the OFC model has recently been recognized as an active educational approach in various fields because researchers and teachers have shown interest in this strategy [24–26].

Flipped classrooms increase the flexibility of teaching and change the role of teachers and learners [27]. Learners’ role is shifted from inactive to active, and instructors’ roles change from leader to supporter and mentor [28]. Learners also have more opportunities to participate and interact with instructors due to broader access to resources [29]. A systematic review by Tan et al. (2017) showed that this strategy helps learners improve knowledge, skills, self-learning, learning satisfaction, critical thinking, and problem-solving skills [28]. Online learning can cover a range of applications, systems such as computer-based learning and web-based learning. One of the computer applications to learning is the Learning Management Systems (LMS) [30], and it is often used to prepare a flipped classroom [31].

Given the need to support learners’ participation and strengthen their self-directed learning skills, especially in distance education [32], the lack of an effective teaching method during the COVID-19 pandemic, and insufficiency of studies on the effectiveness of various online education methods, including flipped classroom, especially online courses during the COVID-19 pandemic [6, 21], and the supportive role of social media, the present study was conducted to determine the effect of the flipped classroom along with online teaching on self-directed learning and metacognitive awareness of nursing students. Previous studies focused on online asynchronous (OA) Learning (which allows learners to watch instructional materials at any time they want and does not include a live video lecture) rather than a combination of online learning methods, which remains a controversial question [21].

## Methods

### Study design

This study was a quasi-experimental single-group pretest-posttest study using intragroup comparison. Since only one group of students is admitted each semester, a group of undergraduate students was selected as both the control group and the intervention group.

### Participants and setting

This study recruited 34 nursing students at Khorramabad School of Nursing and Midwifery, affiliated with Lorestan University of Medical Sciences in Western Iran. The theoretical course of Adults and Older Adults Nursing II on Respiratory, Cardiovascular, and Kidney Disorders were presented in the first semester of the 2020-2021 academic year, simultaneously with the outbreak of the COVID-19 pandemic, and the practical part of the course was presented one semester later. Hence, students did not have the experience of dealing with such cases and clinical care for them. Simultaneously with this course, other courses were mainly held asynchronously (mainly in PDF and podcast files) via LMS.

Nursing students were selected via convenience sampling for this study based on the inclusion criteria of enrollment in the theoretical course of Adults and Older Adults Nursing II and studying in the second year of nursing. Unwilling students and those with previous flipped classroom experience or other self-directed methods were excluded from the study. Since there was no other group of students simultaneously studying with the case group, the participants were considered both the control and intervention groups. The first eight weeks were held, with 34 students receiving OA training as the control group. The same 34 students continued the course online without face-to-face sessions as the OFC group for eight weeks. Finally, the participants were compared with themselves.

### Tools

Data were collected using a socio-demographic information checklist (including age, gender, place of residence, marital status, and active learning experience such as a flipped classroom), the Self-Directed Learning Readiness Scale for Nursing Education (SDLRSNE), and the Metacognitive Awareness Inventory (MAI).

Fisher developed SDLRSNE in Australia in 2001 specifically for nursing [33] with 40 items and three dimensions of self-management (items 1 to 13), desire for learning (items 14 to 25), and self-control (items 26 to 40). Items are scored based on a 5-point Likert scale from strongly disagree (=1) to strongly agree (=5), and the mean total score ranges between 40 and 200 [34]. Four items are scored inversely. A mean score above 150 indicates self-directed learning readiness [35]. The psychometric evaluation of SDLRSNE in Iran reported the Cronbach’s alpha coefficient of 0.82 for the whole scale and 0.60-0.78 for its subscales [36]. In the present study, Cronbach’s alpha for the whole scale and the subscales of self-management, desire for learning, and self-control were 0.94, 0.88, 0.87, and 0.88, respectively.

The following tool was MAI, consisting of 52 items to evaluate the various dimensions of metacognition. Participants respond to each item based on a 7-point Likert scale from strongly agree (=1) to strongly disagree (=7). Its maximum score is 364, and its minimum score is 52 [37]. This tool has already been translated into Persian and psychometric by Kooshki and Shavandi (2019) in Iran. The reliability of the instrument was confirmed by the internal consistency method with Cronbach’s alpha 0.82. In order to evaluate the construct validity, exploratory factor analysis was used. After confirmatory factor analysis, the instrument showed an acceptable fit [38].

### Measuring variables

The link to the data collection tool was sent to the students via WhatsApp™ messenger to observe social distancing and avoid in-person classes in three stages: before uploading the content of the first session and one week before the first lecture (pre-test), at the end of week 8 (post-test 1), and the end of week 16 (post-test 2), and they were asked to complete and submit the tool within a maximum of one week. Before sending the link, the students were briefed on how to complete the tool via the WhatsApp™ channel. The instructions for completing the tool incorporated with the online tool also clearly stated how to complete the tool.

### Intervention

The study began after obtaining permission from the Research and Technology Deputy of Lorestan University of Medical Sciences and coordination with professors. One week before the first lecture, the study tools were completed by the participants. The professors presented the Adult and Older Adults Nursing II course in 16 two-hour sessions, including renal and cardiovascular diseases nursing, divided into two 8-session parts. Eight sessions on renal diseases were presented during the first eight weeks. Eight sessions related to cardiovascular diseases were presented with OFC from mid-semester to the end of week 16 (Table 1).

**Table 1:** Titles of online learning modules for the adults and older adults nursing II course

Sessions groups	OA educational content	OFC educational content
Module 1	Evaluation of kidney and urinary tract diseases and nursing care in diagnostic tests	Management of patients with chronic heart failure
Module 2	Prevention and management of kidney and urinary tract infections	Management of patients with coronary artery disease (angina pectoris)
Module 3	Prevention and management of urinary tract stones	Management of patients with coronary artery disease (myocardial infarction)
Module 4	Nursing care in renal parenchymal diseases (e.g., nephrotic syndrome)	Structural, infectious and inflammatory disorders of the heart (cardiomyopathies)
Module 5	Vital care in surgery and trauma to the kidneys and urinary tract	Structural, infectious and inflammatory disorders of the heart (valvular disorders)
Module 6	Reproductive health and management of infectious diseases of the female reproductive system	Structural, infectious and inflammatory disorders of the heart (infectious heart diseases)
Module 7	Reproductive health and management of infectious diseases of the male reproductive system	Evaluation and care of patients with hypertension
Module 8	Nursing care in male and female genital malignancies	Evaluation and management of patients with vascular disorders and peripheral circulatory problems

All the participating students were briefed regarding the outline, general and specific objectives, and titles and number of sessions of the course, students’ assignments during the course, OFC teaching method, class time (a two-hour module per week), and evaluation and class rules in online sessions in a 90-minute session. They were also uploaded in the references section and the Course Introduction to the local LMS (special software for university learning) to be permanently accessible to the learners who wanted to review the course schedule.

### Teaching methods in the flipped classroom

At the end of the eighth session, the link to the data collection tools was sent to the students’ mobile phones via WhatsApp™ so that they could complete the tools. From the ninth session onward, eight modules on cardiovascular disease management were presented following OFC principles, including a three-step process (pre-class, in-class, and post-class activities), described in detail below.

### Pre-class activities

At the self-learning stage, video or textual contents designed by the instructor were presented outside the official classroom time. To this end, one week before each module was presented, its educational content was made available to the participants in narrated PowerPoint files, podcasts, or short educational videos. One of the instructors, the second author, produced the 20- to 30-minute electronic content by content development standards and with the help of Storyline software. Evidence-based tips by Persky et al. (2017) were also used in designing the content (video or reading material) [39]. Each content included a topic, general and specific objectives, text, summary, and a self-assessment. The online learning platform for content sharing was the interactive LMS of Iran Virtual University of Medical Sciences, accessible at “https://lumsnavid.vums.ac.ir”. These software systems deal with the application, registration, management, follow-up, evaluation, presentation of programs, interactions between learners and program content, and interactions between learners and professors. The content uploaded on this system can be easily viewed through students’ laptops, computers, or mobile phones. Students had access to the content, assignments, self-assessments, discussions, and messages through the platform. Due to the user-friendliness of this platform and students’ experience with it, they knew how to use it. In addition, instructions were available in both video and PDF formats on the platform.

One educational module was accessible to students each week to analyze. Students were asked to carefully and individually read the contents for independent thinking and reflect on them. After the individual study, some students were selected in a maximum of 2-3 groups of 6 individuals each week to do group homework. One student was selected as the coordinator of each group. We tried to have a balanced combination of strong and weak students in each group. At this stage, one of the active learning strategies, such as reflection paper, concept map, and Cornell notes, was used by groups in each session to summarize the content and report group activities. As a pre-assessment, students were asked to discuss a few critical questions about each content one week before the start of the class, focusing on their knowledge, ambiguities, challenges, and previous experiences on the topic in the LMS Discussion Forum.

Moreover, a WhatsApp group was formed to reduce student stress. Teachers were also added to the group. When the need arose and in the case of questions, the teachers answered the questions and monitored out-class discussions. There were also discussions about the content uploaded in LMS and related assignments between students on this channel. All the coordination required for each session was already given in WhatsApp.

### In-class activities

This stage, also known as a dialogue with others, focused on peer-to-peer activities and the online discussion of teachers with students, which lasted about 90 min. After seeing the required training materials, students attended online classes through the Adobe Connect platform at the time specified in the class schedule. At this stage, students spend their time on activities such as problem-solving or discussion with peers. Most students were randomly asked about the subject for a basic learning assessment during this stage, and in-class quizzes were given. Students’ responses were also reviewed and criticized by their peers in the form of a mini-review. Students could share their comments by typing in the chat box or activating the microphone and speaking. They could answer the quizzes via Google Docs™. These activities provided an opportunity to assess and identify students’ learning difficulties.

 Furthermore, during the class, the coordinator of each group prepared a summary of the content of each session as an audio file of 5 min, text, or both at a pre-determined time and shared it in the discussion forum after the students agreed. Other students read the summary and submitted their comments. After presenting the students’ comments, the first author (TA) had to review the comments on a daily and continuous basis and encourage the students to participate as much as possible. At the end of the class, the instructor corrected the students’ misconceptions, provided feedback, and summarized and highlighted essential points related to the content or patient care in the form of microteaching. Students were informed that unlike the initial online class quiz, which did not affect the final evaluation, active participation in the discussion forum and the number of posts (after a qualitative review, of course) impacted the final grade.

### Post-class activities

Extended assignments (descriptive and problem-based) were presented after completing each educational content and entering the following to consolidate learning. Assignments were uploaded in the assignments section of LMS and reviewed by the second author, who provided feedback within a set deadline (about two weeks).

### Learning assessment

Three instructors made the assessment. The tests related to the assessment of essential learning did not have any impact on the final assessment score, while the students’ discussions in the forum and individual and group assignments were considered. Hence, the final evaluation was based on the final exam consisting of multiple-choice questions (40%), group summarization of the content (20%), participation in the discussion forum and online classes (20%), and final projects (20%).

### Teaching methods in the control group

Education was provided as OA for eight consecutive sessions per week. The content of lessons was uploaded in LMS in narrated PowerPoint™ files and podcasts according to the course schedule and its chronological order. Students experienced self-learning by relying on the uploaded content.

### Data analysis

Data were analyzed using Stata-14 software. Descriptive statistics including frequency, percentage, mean and standard deviation were used. Paired t-test was used to compare the mean scores of metacognitive awareness and self-directed learning in the pre-and post-test stages and comparison of the two methods.

## Results

Thirty-four nursing students, including 20 males (59%) and 14 females (41%), participated in this study. Twenty-four (71%) of them were local (living in Lorestan province). Their mean age was 22 (SD=1), and only 2 (6%) of them were married. None of them had the experience of attending flipped classrooms (in person or online).

There was no significant difference between the mean scores of metacognitive awareness (*p*=0.15) before and after implementing the offline method. However, there was a significant difference between the mean score of self-directed learning readiness (*p*=0.0004) before and after implementing the OA method, which indicated an increase. The mean scores of desire for learning (*P*-value=0.01) and self-control (*P*-value=0.02) increased significantly after the OA method (Table 2).

**Table 2:** Mean score of metacognitive knowledge and self-directed learning of nursing students in the OA method

	Total number=34	Total number=34		
	Pre-test	Post-test	Paired t-test of the participants	
	Mean (SD)	Mean (SD)	T	*P*-value
Total score of metacognitive awareness	255.68 (53)	262.68 (48.16)	1.46	0.15
Total score of self-directed learning	148.76 (23.06)	155.82 (20.6)	3.96	<0.001
Self-management	45.5 (8.19)	46.44 (9.1)	0.76	0.45
Desire for learning	45.76 (7.1)	48.18 (6.74)	2.49	0.01
Self-control	58.32 (8.89)	61.03 (8.12)	2.5	0.02

After applying OFC, students’ mean metacognitive awareness and self-directed learning scores confirmed their significant improvement before the intervention. After applying OFC, the mean scores of self-directed learning subscales, including self-management, desire for learning, and self-control, confirmed the significant improvement of the variables compared to before the intervention (Table 3).

**Table 3:** Mean score of metacognitive knowledge and self-directed learning of nursing students in the OFC method

	Total number=34	Total number=34		
	Pre-test	Post-test	Paired t-test of subjects	
	Mean (SD)	Mean (SD)	T	*P*-value
Total score of metacognitive awareness	255.68 (53)	272.03 (50.03)	2.29	0.03
Total score of self-directed learning	148.76 (23.06)	162.03 (21.77)	3.70	<0.001
Self-management	45.5 (8.19)	50.56 (9.48)	4.48	≤0.0001
Desire for learning	45.76 (7.1)	48.94 (7.59)	2.42	0.02
Self-control	58.32 (8.89)	62.5 (7.66)	2.96	<0.01

According to Table 4, the comparison of the mean score changes of both methods indicated a lack of a significant difference between the mean total score of metacognitive awareness (*P*-value=0.15) and the mean total score of self-directed learning (*P*-value=0.07) after the implementation of the OFC and OA methods. Furthermore, the comparison of changes in the mean score of variables between the two methods showed a significant increase in the mean total score of self-management (*P*-value=0.02), while there was no significant difference in the mean total score of desire for learning (*P*-value=0.52) and self-control (*P*-value=0.28).

**Table 4:** A Comparison of the mean scores of metacognitive knowledge and self-directed learning of students between the two methods

	Total number=34	Total number=34		
	OA method	OFC method	Paired t-test of subjects	
	Mean (SD)	Mean (SD)	t	*P*-value
Total score of metacognitive awareness	262.68(48.16)	272.03(50.03)	-1.49	0.15
The total score of self-directed learning	155.82(20.6)	162.03(21.77)	-1.88	0.07
Self-management	46.44(9.1)	50.56(9.48)	-2.36	0.02
Desire for learning	48.18(6.74)	48.94(7.59)	-0.66	0.52
Self-control	61.03(8.12)	62.5(7.66)	-1.1	0.28

## Discussion

The present study showed that the mean total score of self-directed learning readiness increased after using both methods (OA and OFC), and as a result, they positively affected students’ desire for learning and self-control, but there was no significant difference between the two groups. Among the self-directed learning subscales, the mean score of the self-management variable increased in the OFC method.

Evidence suggests that synchronous and asynchronous activities are both critical and complementary because a synchronous approach increases learners’ incentive and attention, and an asynchronous approach increases data processing ability [21]. Some sources refer to the asynchronous method as self-paced learning, which leads the learner to self-direction [40]. It occurs when learners perform learning activities in proportion to their speed by having the right to choose the task time [13]. Traditional online courses increase flexibility by focusing on asynchronous tasks [21]. The OA method also showed positive results for the self-directed variable could be attributed to learners’ high motivation. In the absence of instructors and classmates in the OA method, being motivated is the main incentive whose absence will make students’ involvement with the subject and learning more difficult.

On the other hand, OFC based on an LMS also positively affected learners’ readiness for self-directed learning. According to Ugwoke (2018), flipped classrooms via online tools can develop students’ interest in learning and meet learners’ needs. In the flipped classroom using LMS, students can freely choose the most proper ways to obtain new knowledge [41]. Learners can develop self-directed and self-paced learning skills by studying the content prepared by the teacher asynchronously before starting the class. Self-management skills can be developed using the content along with the teacher’s supervision [13]. Before entering the flipped classroom, mastery of the content increases learners’ intuitive learning skills and confidence to perform classroom activities [42]. Studies have also shown that flipped classrooms improve learners’ self-directed learning readiness and increase their satisfaction [18]. There is evidence suggesting that the flipped classroom approach positively affects learners’ motivation and significantly improves self-directed readiness skills [18, 20, 43]. Stoher et al. (2020) compared the effect of OFC with campus-based courses and reported no difference in the average performance between the two groups. In their study, OFC was compared with campus-based courses [21]. Also, postgraduate students participated in their study. There is emerging evidence that age and previous education may positively affect self-directed learning readiness [33].

One of the internal factors affecting self-directed learning is the readiness to accept the responsibility of learning, and that constitutes the two main characteristics of the flipped classroom and autonomy [44]. Furthermore, self-directed learning readiness is like a cycle in the learning process that requires prior readiness and motivation to enter this cycle [45]. Students were well prepared given the average score of learners’ self-directed learning readiness before the intervention. Another possible explanation is that the teachers of the studied faculty (including the second and third authors) had used new and active educational methods, particularly e-learning and blended education, to teach their students for many years, including the participants in this study, and that they had positive experiences in teaching the same students with a blended method from the previous semester. Students’ previous experience using e-learning and blended learning gave them high self-confidence to take responsibility for their learning and thus achieve good self-direction readiness. Zamnah (2019) et al. showed that learning self-direction increases students’ self-confidence [46].

An essential feature of self-directed learners is that they make good use of online learning environments [47], and at the same time, scaffolding should be considered, as seen in this study and following the methods mentioned above. Robinson et al. (2020) believe that the development of self-directed learning requires a scaffolding approach such that teacher-centered activities help learners achieve more self-regulation in self-direction, for which flipped classroom is suggested [13]. In the self-directed learning process, learners set goals, evaluate their progress, structure activities over time, identify resources, and seek feedback [13]. The body of evidence also suggests that self-directed learning readiness in online environments leads to better academic performance and learners’ success. In particular, online environments provide learners with the right to choose how to learn to strengthen self-directed readiness [48].

LMS promotes teaching and learning as a portal to innovative technology and provides forum discussion and several interactive features [49]. Adind et al. (2020) stated that discussion forums allow learners to interact, encounter, and actively process problems. Therefore, it promotes their learning, which they believe results from the development of students’ self-directed learning [48]. Moreover, in a well-designed SDL environment, motivation, a sense of control, self-confidence, and self-belief increase [13]. The results of the present study results showed that OFC had improved self-management in learners compared to the OA method. Self-management is vital in self-directed learning, and learners should choose different strategies to deal with challenges [50].

The results of the present study also indicated that the OFC method promotes the metacognitive awareness of learners. There is a growing body of evidence to suggest that the OFC method promotes the metacognitive awareness of learners [19, 51, 52].

Chan et al. (2021) conducted a similar study to determine the effectiveness of active learning (blended flipped classrooms and enhanced lectures) on the improvement of the metacognitive awareness of students, the results of which were consistent with the present study. According to the authors, flipped classrooms make students take responsibility for their learning. As a result, they will have more flexibility to learn. [53]. Metacognitive awareness and independence in learning are among the skills necessary for distance learning, which require much time to acquire and cannot be quickly learned through distance learning methods [54]. Also, independence in learning requires achieving metacognitive awareness. Metacognitive skills such as metacognitive awareness control and regulate cognitive processes through independence in learning and self-direction [55]. In this study, according to students’ long-term distance learning experiences in previous semesters, metacognitive awareness improved with OFC despite the short intervention time. According to Bektas (2020), metacognitive awareness increases students’ self-confidence and motivation, such that increasing self-confidence will help them learn with their advanced mechanisms and use appropriate opportunities to access resources and information. Eventually, they achieve higher metacognitive awareness [56, 57]. In the present study, the mean total score of students’ metacognitive awareness before the intervention increased their self-confidence and motivation. Siqueira (2020) also reported a positive relationship between metacognitive skills and motivation in those active methods such as flipped classrooms could motivate and ultimately develop metacognitive skills [45].

Furthermore, metacognitive skills are part of self-directed learning [57]. Ultimately, self-directed learning in active approaches such as flipped classroom develops metacognitive skills, including metacognitive awareness [53]. Mukaddes Örs et al. found a positive and significant correlation between self-directed learning in nursing students [57].

Moreover, in this study, we tried to use WhatsApp in parallel with LMS to strengthen the OFC. Arifani et al. (2020) realized that this strategy could be an excellent way to promote the cohesive ability of English learners [58]. One of the most favorite applications is WhatsApp and learners should be encouraged to integrate informal learning activities to support and increase formal learning [59]. During the Covid-19 outbreak, online learning environments became critical, particularly teacher-student interaction in these contexts [60]. The teaching method was suddenly changed to a completely online method due to the COVID-19 epidemic. Because we did not have access to the students for face-to-face communication, they were not sufficiently prepared for the intervention. However, before the intervention, the students were briefed regarding their duties and expectations using WhatsApp™ messenger to prepare them for the interventions.

Our study had some limitations. It was a quasi-experimental and single-group study. At our university, only one group is accepted each semester. Also, the sample size was small. It is recommended that further studies with a larger sample size be performed as a clinical trials with a longer course in the future.

## Conclusions

With the increasing growth of online learning during the COVID-19 pandemic and the effectiveness of the flipped classroom as a novel method, this study was conducted to determine the effectiveness of OFC on self-directed learning readiness and metacognitive awareness of nursing students. The results of the present study results showed the effectiveness of LMS-based online learning methods on students’ self-directed learning readiness and metacognitive awareness. Students had good self-direction and metacognitive knowledge before the intervention and had the experience of learning with distance and hybrid teaching methods. This background, combined with the active educational approach used, yielded satisfactory results despite the short duration of the intervention. Hence, further studies with more extended periods are recommended to examine the effect of OFC on self-directed learning and other related variables.

## Data Availability

Data and materials are available at the request of the readers. Requests for data should be addressed to the corresponding author.

## Abbreviations

OFC: Online flipped classrooms
LMS: Learning Management Systems
OA: Online asynchronous
SDLRSNE: Self-Directed Learning Readiness Scale for Nursing Education
MAI: Metacognitive awareness inventory
TA: Teacher assistant
